# Angiogenic properties of aged adipose derived mesenchymal stem cells after hypoxic conditioning

**DOI:** 10.1186/1479-5876-9-10

**Published:** 2011-01-18

**Authors:** Anastasia Efimenko, Ekaterina Starostina, Natalia Kalinina, Alexandra Stolzing

**Affiliations:** 1 Department of Biological and Medical Chemistry, Faculty of Fundamental Medicine, Lomonosov Moscow State University, Moscow, Russia; 2 Fraunhofer Institute for Cell Therapy and Immunology, Leipzig, Germany

## Abstract

**Background:**

Mesenchymal stem cells derived from adipose tissue (ADSC) are multipotent stem cells, originated from the vascular-stromal compartment of fat tissue. ADSC are used as an alternative cell source for many different cell therapies, however in ischemic cardiovascular diseases the therapeutic benefit was modest. One of the reasons could be the use of autologous aged ADSC, which recently were found to have impaired functions. We therefore analysed the effects of age on age markers and angiogenic properties of ADSC. Hypoxic conditioning was investigated as a form of angiogenic stimulation.

**Methods:**

ADSC were harvested from young (1-3 month), adult (12 month) and aged (18-24 month) mice and cultured under normoxic (20%) and hypoxic (1%) conditions for 48 h. Differences in proliferation, apoptosis and telomere length were assessed in addition to angiogenic properties of ADSC.

**Results:**

Proliferation potential and telomere length were decreased in aged ADSC compared to young ADSC. Frequency of apoptotic cells was higher in aged ADSC. Gene expression of pro-angiogenic factors including vascular endothelial growth factor (VEGF), placental growth factor (PlGF) and hepatic growth factor (HGF) were down-regulated with age, which could be restored by hypoxia. Transforming growth factor (TGF-β) increased in the old ADSC but was reduced by hypoxia.

Expression of anti-angiogenic factors including thrombospondin-1 (TBS1) and plasminogen activator inhibitor-1 (PAI-1) did increase in old ADSC, but could be reduced by hypoxic stimulation. Endostatin (ENDS) was the highest in aged ADSC and was also down-regulated by hypoxia. We noted higher gene expression of proteases system factors like urokinase-type plasminogen activator receptor (uPAR), matrix metalloproteinases (MMP2 and MMP9) and PAI-1 in aged ADSC compared to young ADSC, but they decreased in old ADSC. Tube formation on matrigel was higher in the presence of conditioned medium from young ADSC in comparison to aged ADSC.

**Conclusions:**

ADSC isolated from older animals show changes, including impaired proliferation and angiogenic stimulation. Angiogenic gene expression can be partially be improved by hypoxic preconditioning, however the effect is age-dependent. This supports the hypothesis that autologous ADSC from aged subjects might have an impaired therapeutic potential.

## Introduction

Mesenchymal stem cells (MSC) have therapeutic potential in bone marrow transplantation [1,2], tissue engineering [3], and cell therapy [4]. Adipose-derived stem cells (ADSC) are relatively easy to obtain from adipose tissue and are more frequent than MSC in bone marrow [5]. They represent therefore a promising source for cell therapy, especially as their isolation is less invasive compared to bone marrow extractions and their expansion in culture is quite easy [6,7]. The use of MSC from aged bone marrow donors have been investigated and found to be less effective in myocardial infarction treatment in a mouse model probably because of age-induced changes [8]. For bone marrow derived MSC several studies analysed age-related changes [9] which were possibly responsible for the impaired therapeutic impact.

It is shown that ADSC are able to differentiate into the classical mesodermal tissues like bone, fat and cartilage [10], and it is claimed that they can differentiate into nerve, cardiomyocytes, hepatocytes and pancreatic cells [11,12]. ADSC show the same surface markers as bone marrow derived MSC [13,14]. For either cell type it is not clear if a small subpopulation of the MSC might contain additional differentiation capacity [15]. The in vivo potential of these cells is unclear, however support of neuronal repair [16], osteogenesis [17] and vasculogenesis were shown [18]. In some studies it was demonstrated that ADSC could release multiple angiogenic growth factors and cytokines/chemokines. This suggest that they may have potential as a useful cell source for therapeutic angiogenesis [19].

Upon in vitro expansion it was shown that ADSC age after about 30 population doublings [20] losing adipogenic differentiation capacity [21]. One group reported spontaneous transformation of ADSC. However no tumors were observed after injection of the cells into immune-compromised mice [22]. To minimise in vitro aging effects ADSC and other stem cells have been cultured under reduced oxygen concentrations with mixed results [23,24]. Differentiation of ADSC seems to be supported by higher oxygen levels and expansion by low oxygen levels [25].

Aging negatively affects angiogenesis which is found to be linked to declined levels of VEGF after ischemic stimuli [26]. Therefore, aging-associated changes may constitute a link to cardiovascular diseases and stroke in the elderly [27]. ADSC are thought to mediate angiogenesis by releasing growth factors including VEGF, HGF and basic fibroblast growth factor (bFGF). These factors stimulate endothelial cell division, migration, stromal progenitor cell grafting into the forming vessels. They also facilitate mobilization of bone marrow endothelial precursors which participate in neovascularization [28,29]. It is a matter of ongoing discussion whether this property is an important part of the regenerative mechanism of ADSC and is under investigation in several pre-clinical trials [30-33]. As angiogenesis seems to be an important factor in tissue remodelling in cell therapies we investigated the changes of the angiogenesis-related factor production in aged ADSC.

Hypoxia is known to stimulate the pro-angiogenic effects of ADSC [28,34], but little is known about the reactivity of aged ADSC upon a short term exposure to hypoxia. We therefore investigated ADSC from young and aged mice under low oxygen. The exact concentration of oxygen in adipose tissue is not known and we therefore used the levels of oxygen presumed for the bone marrow niche [24]. Proliferation and osteogenic differentiation was therefore tested for ADSC cultured under normoxia (20%) or hypoxia (1%) similar to levels found to be beneficial for bone marrow derived MSC.

## Materials and methods

### Chemicals

All chemicals were obtained from Sigma-Aldrich unless stated otherwise.

### Isolation of adipose derived mesenchymal stem cells

C57/Black6 mice were obtained from the University of Leipzig (Approval for schedule 1 sacrifices were given by the Regierungspräsidium Leipzig, Germany). ADSCs were isolated from adipose tissue of mice as described [6,10,12]. Adipose tissue was washed with PBS, minced and treated with 200 U/ml collagenase I in DMEM for 1 h at 37°C. Tissue was centrifuged at 1200 g for 5 min and the supernatant discarded. The pellet containing the ADSC was lysed to destroy erythrocytes. The material was then filtered through a sieve, and washed. The cells were cultured at normoxic conditions (20% of oxygen) until the experiment starts.

### Hypoxia

The day before hypoxia establishment the medium was replaced with serum-free medium. Then cells have been cultivated for 48 h under hypoxia (1% of oxygen) or normoxia (20% of oxygen) conditions. Parameters of hypoxia modelling were chosen as the most favourable for inducing changes in gene expression in bone marrow derived MSC [35,36]. Cell properties were analyzed immediately after cells had been taken off the hypoxic incubator.

### Cell viability assessment

The number of early apoptotic and dead cells was evaluated with flow cytometry by phycoerythrin conjugated annexin V (AnnexinV-PE) binding and 7-amino-actinomycin (7-AAD) dye accumulation. Cells were removed from culture plate surface by EDTA in PBS (2 mM, 5 min, 37°C). 100 μl of cell suspension (1-10 × 10^5 ^cells/ml) were incubated with AnnexinV-PE for 20 min and washed with buffer according to the manufacturing instructions. Then 7-AAD was added for 5 min. Stained cells were analyzed with cell sorter MoFlo (Dako Cytomation). Cell number at various apoptosis stages (AnnexinV^+ ^7-AAD^-^) and number of viable (AnnexinV^- ^7-AAD^-^) and dead cells (AnnexinV^+ ^7-AAD^+^) were calculated.

### Immunophenotyping of ADSC

Cells were harvested by trypsinisation and stained with antibodies against CD14, CD19, CD34, CD45, CD73, CD79, CD90, CD105, NG2 and PDGFRB (diluted 1:100; 4°C; 30 min; Serotec, Oxford, UK). The cells were analysed using flow cytometry. CD11b antibody (1:100; 4°C; 30 min; Serotec, Oxford, UK) was used as a negative control.

### Proliferation and osteogenic differentiation of ADSC

Cells were grown in 24 wells at 20,000 ADSC per well. Proliferation was measured using MTT test (Invitrogen). For osteogenic differentiation 40,000 ADSC were seeded in 24 well plates with medium containing 10^-8 ^M dexamethasone and 5 μg/ml ascorbic-2-phosphate for 14 days. Alkaline phosphatise (ALP) quantitation was performed using a colorimetric assay using ρ-nitrophenyl phosphate (ρ-NPP) as substrate [37]. Ethanol-fixed cells in 24-well plates were reacted with 200 μl of ALP assay buffer (5 mM ρ-NPP, 0.5 mM MgCl2, 0.1% Triton X-100, and 50 mM TBS, pH 9.5). After incubation at room temperature for 10 min, the absorbance was measured at 405 nm. The cells were washed, and cell number was determined.

### Reactive oxygen species (ROS) and nitric oxide (NO)

Cells were incubated in the dark with 5-(and-6)-chloromethyl-2,7-dichlorodihydrofluorescein diacetate acetyl ester (H_2_DCF-DA) (50 μM) for 30 min, washed and analysed with a flow cytometer. Nitric oxide concentration was measured using standard Griess reagent. 50 μl supernatant was incubated with 50 μl Griess reagent. After 5 min incubation at RT, the absorbance was measured at 560 nm using a tecan plate reader.

### Real-time PCR

RNA was isolated according to instructions (RNA/DNA/Protein Purification Kit, Norgen, Canada). RNA concentration was measured using the NanoDrop (Peqlab). One μg of total RNA was used to synthesize cDNA with SuperscriptIII-transcriptase (70 U/1 μg RNA; Invitrogen) and Oligo(dT)18-Primers (Fermentas, see Table 1 for the different primer sequences used). For qRT-PCR (LightCycler 480, Roche) the cDNA was diluted 1:10 and 5 μl added to Express SYBR GreenER qPCR Supermix Universal (Invitrogen) Serial dilutions of recombinant standard DNAs containing the analyzed gene sequences served as controls. Transcript amounts are normalized to GAPDH, 18S or L7.

**Table 1:** PCR primers used

Gene	Sense strand	Antisense strand
c-myc	AGTGCTGCATGAGGAGACAC	GGTTTGCCTCTTCTCCACAG
p53	TCCCCCGCAAAAGAAAA	CTGTAGCATGGGCATCCTTT
oct-4	ACATGAAAGCCCTGCAGAAG	AGATGGTGGTCTGGCTGAAC
18s	CAGTAAGTGCGGGTCATAAGC	ATCCGAGGGCCTCACTAAAC
VEGF-a	AGAGCAGAAGTCCCATGAAGTGA	TCAATCGGACGGCAGTAGCT
PlGF	CCAAGGGGAAGAGGAAGAGGAGTA	GCAGGGACGAGTCGGCTAATAA
bFGF	GCGCCGCCTTCCCACCAG	AGCCAGCAGCCGTCCATCTTCCT
HGF	TCATTGGTAAAGGAGGCAGCTATA	CTGGCATTTGATGCCACTCTTA
TGFb	TGCCCCTATATTTGGAGCCTGGAC	GCCCGGGTTGTGTTGGTTGTAGAG
uPA	GAATGCGCCTGCTGTCC	AGGGTCGCTTCTGGTTGTC
uPAR	CGTTACCTCGAGTGTGCGTCCTG	AGCCTCGGGTGTAGTCCTCATC
MMP2	AGTTCCCGTTCCGCTTCC	AGCCTCGGGTGTAGTCCTCATCC
MMP9	GCGGTGTGGGGCGAGGTG	CCAGGGGGAAAGGCGTGTGC
ENDS	AGTTTGGTCTTGCTGCTGGTG	AAGTCCCGGAAGAAGAGTTTTG
TBS1	GCGCGGAGCTGGATGTA	AATGTCTTCTGGGGTGGTTC
PAI-1	GCTTCATGCCCCACTTCTTCAA	ACCAGGCGTGTCAGCTCGTCTA
L7	CTCCGTCTGCGGCAGATC	CAGCATGTTAATTGAAGCCTTGTT
GAPDH	GACCCCTTCATTGACCTCAACTAC	TGGTGGTGCAGGATGCATTGCTGA

### Quantitative telomerase activity assay

Cell pellets were resuspended in lysis buffer and analyzed with qRT-PCR using the quantitative telomerase detection kit (QTD, Allied Biotech Inc.). Protein concentration was determined with the BCA Protein assay (Pierce). Assays were conducted with 1 μg of cell extract, using 0,5 μM telomerase primer TS (5'-AATCCGTCGAGCAGAGTT-3') and 0,5 μM anchored return primer ACX (5'-GCGCGG(CTTACC)3CTAACC-3') in 25 μl using 12,5 μl Express SYBR GreenER (Invitrogen). The mix was incubated for 30 min at RT and used in the qRT-PCR. The threshold cycle value (Ct) were determined from semi-log amplification plots and compared with a standard curve generated from serial dilution of telomerase positive HeLa cell extract. Telomerase activity was expressed relative to HeLa cells. The negative control was a heat control incubated for 15 minutes at 85°C.

### Telomere length measurement by quantitative PCR

DNA was isolated using the above mentioned kit (Purification Kit, Norgen, Canada) and concentration was measured using the nanodrop (Peqlap). Telomere length was measured and analyzed by qRT-PCR [38]. PCR was performed in a Light Cycler 480 (Roche).

### Carbonyl-ELISA

Protein carbonyls were measured according to Buss et al. [39] with modifications described by Sitte et al. [40]. The absorbance was determined with a multiwell plate reader using a detection wavelength of 492 nm.

#### GpX activity

Cells were lysed in a buffer and the proteins isolated. After measuring the protein concentration using BCA, GPX activity was measured according to instructions provided by the company (Cayman Chemical).

### In vitro tube formation assay

Effect of young and aged ADSC conditioned medium in vitro on capillary-like tubes formation on Matrigel was evaluated. Conditioned medium was collected from ADSC of young (1.5-2 months old) and aged (18 months old) mice cultured in hypoxic and standard conditions. Human umbilical vein endothelial cells (HUVEC) at the 2-4th passages were seeded in 48-well plates coated with growth factor reduced Matrigel (BD Bioscience) in concentration 2 × 10^4 ^cells per well. HUVEC growth medium was supplemented with conditioned medium in ratio 1:1. At least, two wells were used for each sample of conditioned medium. HUVEC serum-free growth medium was utilized as negative control; HUVEC growth medium with 20% FBS served as positive control. Plates were placed into CO_2_-incubator at 37°C and capillary-like structures were assayed in 24 h under the light microscope (Leica, Germany). Total length of tubular structures was counted in 5 random fields of view (objective 10×) with MetaMorph 5.0 software (Universal Imaging).

### Data analysis

Statistical analysis was performed using SigmaStat 9.0. If normality of data was confirmed (according to the Shapiro-Wilkson test) comparison of independent groups was performed by Student t-test, if not - by one-way analysis of variance (Cruscal-Wolles ANOVA) and Mann-Whitney U-criteria. Multiple comparisons were made using Tukey's test. Values were expressed as mean +/- standard deviation for normally spread data and as median (25, 75 percentile) for not normal data. Data was considered significantly different when p < 0.05.

## Results

### ADSC characteristics

Analyzing of ADCS immunophenotype by flow cytometry showed that cells were positive for CD73 (83 ± 8%), CD90 (>95%) and CD105 (>95%) with no or low expression of CD14 (<10%), CD19 (<10%), CD34 (<5%), CD45 (<1%), CD79 (<10%). This expression profile is normal for MSC according to the International Society for Cellular Therapy Statement of minimal criteria for defining MSC [41]. ADSC also expressed pericyte markers like neural glial antigen (NG2) and platelet derived growth factor receptor beta (PDGFRB), which stayed in agreement with a conception of this type of progenitors as perivascular cells (data not shown) [42,43].

**Figure 1:**
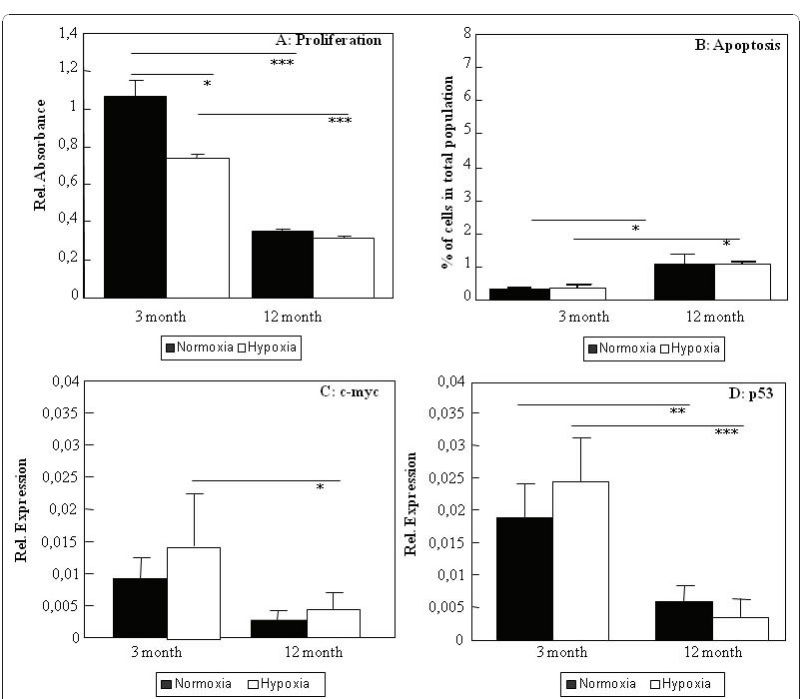
**Proliferation and viability analysis of ADSC**. Proliferation was measured in young and aged (12 month) ADSC cells cultured under normoxia or hypoxia for 48 h before the test (A). Level of cell death was measured using a double staining of 7-AAD and annexinV quantified by flow cytometry (B). Expression of c-myc (C) and p53 (D) were measured using qRT-PCR. Results are means of 5 different animals and considered significantly changed when: * = p < 0.05, ** = p < 0.01, *** = p < 0.001.

ADSC derived from young mice proliferated significantly faster than those from old mice (Figure 1a). Young ADSC under hypoxic conditions (1%) proliferate significantly slower. Aged ADSC under hypoxic conditions did not decrease their proliferation rate (Figure 1a).

Measurements of annexin V binding and 7-AAD accumulation by flow cytometry showed that number of apoptotic cells was 3-fold higher in aged ADSC. Both young and aged ADSC cultivation under hypoxia neither induce cell death by apoptosis nor reduce their general viability (Figure 1b).

**Figure 2:**
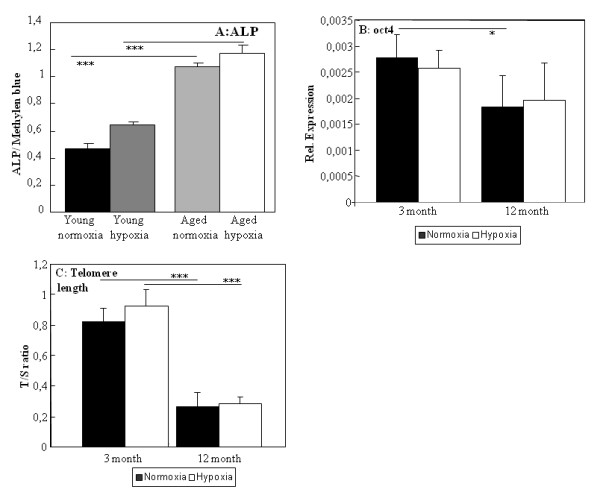
**Osteogenic differentiation, expression of oct4 and telomere length in ADSC**. Ability of ADSC for osteogenic differentiation in the osteogenic induction medium was analyzed using ALP measure (A). Expression of oct4 (B) and changes in telomere length (C) were investigated using qRT-PCR. Results are means of 5 different animals and considered significantly changed when: * = p < 0.05, ** = p < 0.01, *** = p < 0.001.

Cell senescence is accompanied with changes in cell cycle regulators. C-myc is found to be down-regulated in aged ADSC in normal and hypoxic treated cultures (Figure 1c). Surprisingly we find the same decrease in the expression pf p53 (Figure 1d). Analyzing the osteogenic differentiation of ADSC we showed that level of ALP per cell in ADSC cultured in osteogenic induction medium was higher in aged cells compared to young (Figure 2a).

**Figure 3:**
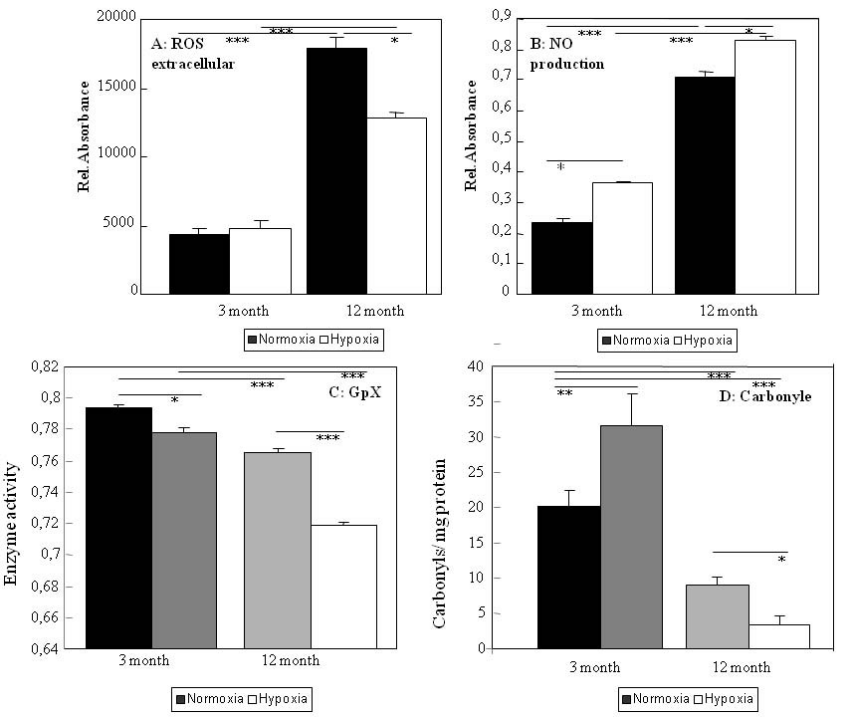
**Oxidative damage of ADSC**. ROS are measured using H_2_DCF-DA staining of ADSC and quantification in a flow cytometer (A). NO concentration was measured using standard Griess reagent (B). Expression level of glutathione dismutase gene was measured using qRT-PCR (C). The level of oxidative damage was investigated using ELISA in young and aged ADSC (normoxia and hypoxia) (D). Results are means of 5 different animals and considered significantly changed when: * = p < 0.05, ** = p < 0.01, *** = p < 0.001.

We measured ROS and NO levels because they are both important signal molecules for osteogenic differentiation of ADSC and a marker of cell quality. ROS levels as well as NO production were significantly increased in aged ADSC (Figure 3a, b). The levels of ROS were significantly down-regulated in aged ADSC under hypoxic conditions; however they were not affected in young ADSC (Figure 3a).

Increasing oxidative stress is linked to induction of differentiation. We therefore measured therefore the level of the stemness gene oct4 which is expressed in MSC. Aged ADSC oct 4 expression is significantly downregulated for normal but not for ADSC under hypoxia (Figure 2c).

The loss of proliferation due to age is often linked to telomere shortening in somatic cells. In ADSC we found a dramatic shortening of telomere length due to the age of the mice. The short term culturing under hypoxic conditions did not affect telomere length (Figure 2c). Increased ROS production might be linked to declining antioxidant activities. Therefore we measured the activity of glutathione peroxide (GPx) activity, arguably one of the most important antioxidative enzymes. GPx activity decreased with age in ADSC. In aged ADSC cultures under hypoxic conditions the activity decreased even further (Figure 3c). Levels of carbonyls were significantly down-regulated in aged ADSC under normoxic and hypoxic conditions (Figure 3d). Interestingly we observed increased carbonyl levels in young ADSC but decreased carbonyl levels in aged ADSC under hypoxia.

### Angiogenesis

Expression of several genes involved in angiogenesis was analysed in ADSC of different age. To evaluate the dynamic of age-changes in gene expression we included an additional group of 24 months old animals.

**Figure 4:**
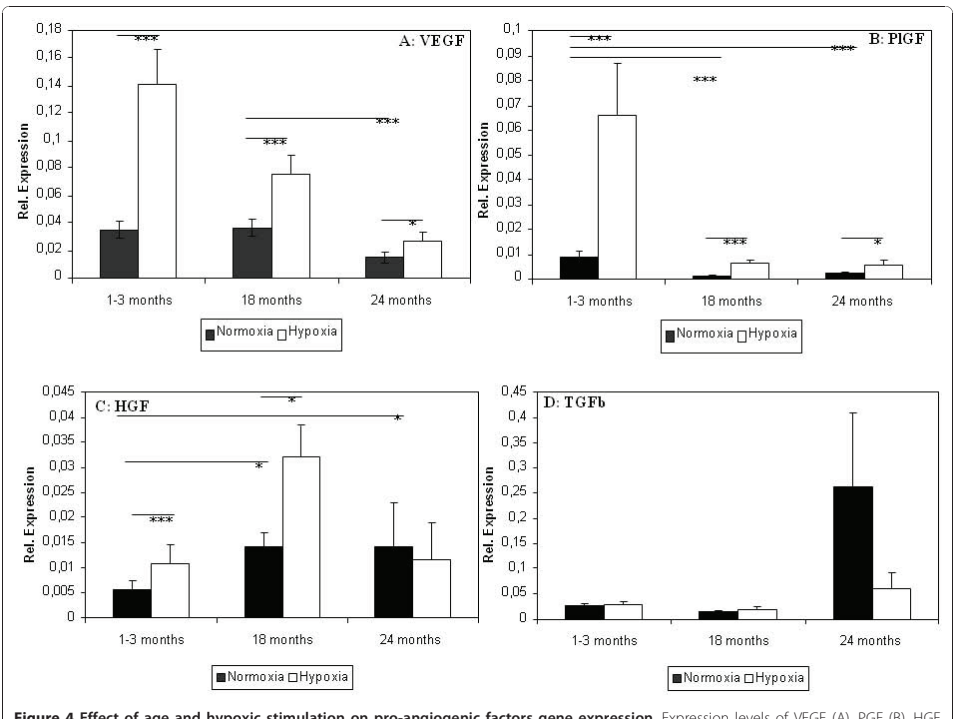
**Effect of age and hypoxic stimulation on pro-angiogenic factors gene expression**. Expression levels of VEGF (A), PGF (B), HGF (C) and TGF-beta (D) were measured using qRT-PCR. Animal groups consisted of young (1-3 month, 16 animals), aged (18 months, 9 animals) and old mice (24 months, 7 animals). Results are means of different animals (* = p < 0.05, ** = p < 0.01, *** = p < 0.001).

Gene expression of VEGF, a pro-angiogenic acting cytokine, declined significantly in ADSC from 24 month old mice and was unchanged in 18 month old mice compared to the 3 month old group. Hypoxia significantly stimulated the production of VEGF in all measured groups; however the effect declined with increasing age (Figure 4a).

PlGF, another pro-angiogenic factor, already was down-regulated significantly in ADSC from 18 month old mice and also in ADSC from 24 month old mice. Hypoxia increased mRNA content of PlGF in all groups. However the size of the changes induced by hypoxia in ADSC declined rapidly with age (Figure 4b).

Interestingly, gene expression of HGF, an important angiogenic and anti-apoptotic factor, was significantly higher in ADSC from 18 and 24 month old mice compared to young ADSC, but aged cells showed worse response to hypoxia, which resulted in the similar level of HGF in young and aged ADSC cultured under hypoxic conditions (Figure 4c).

Gene expression of TGF-β_1 _were similar in ADSC from young and aged animals but did increase significantly in old ADSC (Figure 3d). Hypoxia did not alter expression levels in young or aged ADSC but declined TGF-β_1 _expression in old ADSC.

**Figure 5:**
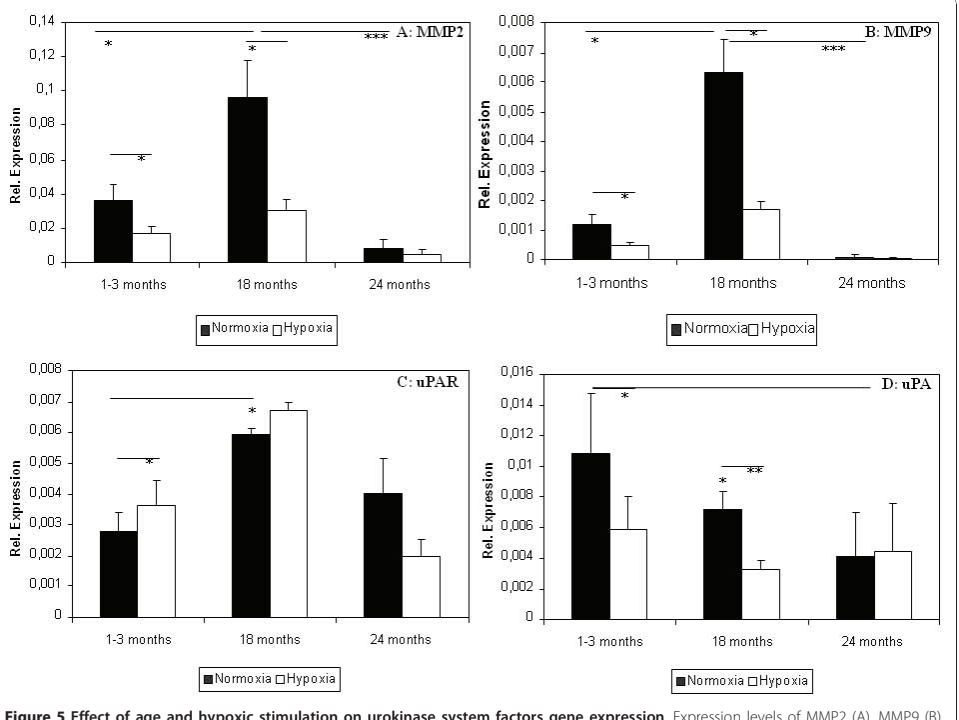
**Effect of age and hypoxic stimulation on urokinase system factors gene expression**. Expression levels of MMP2 (A), MMP9 (B), uPAR (C) and uPA (D) were measured using qRT-PCR. Animal groups consisted of young (1-3 month, 16 animals), aged (18 months, 9 animals) and old mice (24 months, 7 animals). Results are means of different animals (* = p < 0.05, ** = p < 0.01, *** = p < 0.001).

Factors regulating migration and chemotaxis of stem cells like urokinase (uPA) and its receptor (uPAR) as well as MMPs are involved in angiogenesis by degrading the matrix. Expression of MMP2 and MMP9 significantly increased in aged ADSC (18 month old mice) compared to young ADSC but declined in old ADSC. In hypoxic conditions MMPs expression level could be decreased significantly in young and aged ADSC but not in old ADSC (Figure 5a, b). uPAR expression significantly increased in aged ADSC and hypoxia stimulated its expression only in young ADSC (Figure 5c). uPA expression declined significantly in old ADSC compared to young ADSC. Hypoxia inhibited uPA expression in aged ADSC but this effect did not reach significance in young ADSC (Figure 5d). ADSC of the oldest group of mice did not react to the hypoxia stimulus (Figure 5).

**Figure 6:**
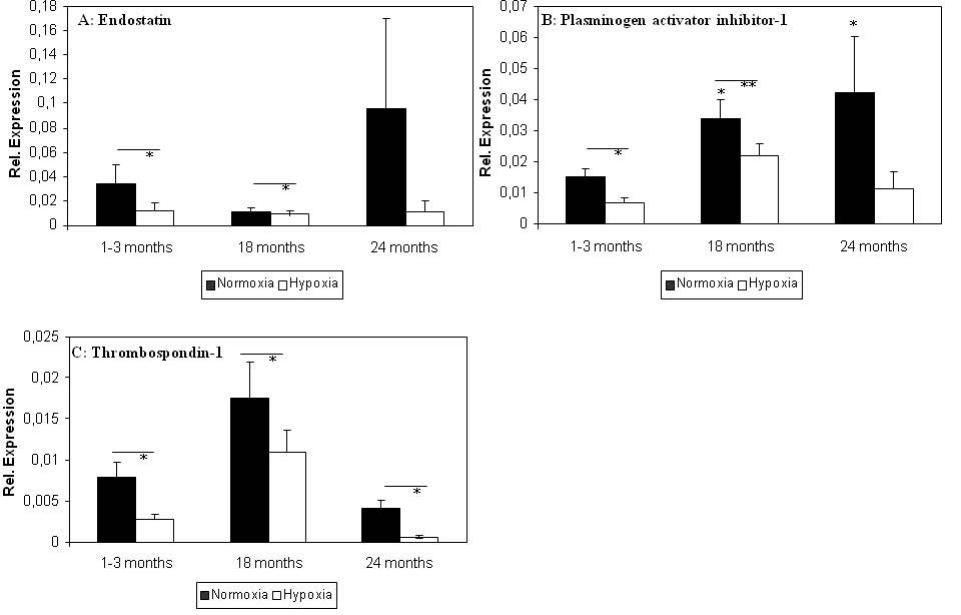
**Effect of age and hypoxic stimulation on anti-angiogenic factors gene expression**. Expression levels of ENDS (A), PAI (B) and TBS1 (C) were measured using qRT-PCR. Animal groups consisted of young (1-3 month, 16 animals), aged (18 months, 9 animals) and old mice (24 months, 7 animals). Results are means of different animals (* = p < 0.05, ** = p < 0.01, *** = p < 0.001).

Expression of anti-angiogenic factors, including PAI-1, ENDS and TBS1 was higher in aged ADSC compared to young ADSC, but the elevation was significant only for PAI-1. Cell culture under hypoxic conditions inhibited expression of all anti-angiogenic factors (Figure 6a, b, c).

**Figure 7:**
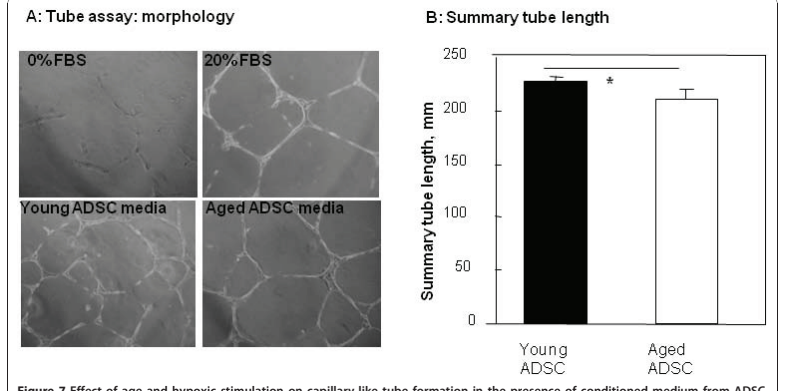
**Effect of age and hypoxic stimulation on capillary-like tube formation in the presence of conditioned medium from ADSC**. Morphology of tubes formed by endothelial cells on matrigel incubated with either medium alone, FBC containing medium or ADSC conditioned medium (A). Summary length of the formed tubes was quantified (B) (* = p < 0.05).

To evaluate the summary of angiogenic factors produced by ADSC we examined the effect of conditioned medium from cells cultured under different conditions on the capillary-like tube formation (Figure 7a, b). Tube formation assay showed that more extensive network of capillary-like structures on Matrigel was formed in the presence of conditioned medium from young ADSC as compared with aged ADSC (p = 0.01). Conditioned medium from both types of cells cultured under hypoxia better stimulated formation of capillary-like structures than the medium from cells cultured under normoxic conditions (p = 0.012 for young ADSC and p = 0.074 for aged ADSC), but difference in this elevation between young and aged ADSC was insignificant (13 ± 3% for young ADSC vs. 11 ± 4% for aged ADSC, p = 0.49).

## Discussion

Adipose tissue derived MSC are a good alternative source to bone marrow derived MSC for cell therapy because of their easy access and high cell numbers. However they seem to undergo similar age-induced changes as bone marrow derived MSC [44]. Here we tested the effect of short-term hypoxic conditioning during cultivation of young and aged MSC from adipose tissue. Hypoxic conditioning has shown beneficial effects in bone marrow derived MSC, increasing their angiogenic properties [36].

We found more apoptotic cells among aged ADSC compared to young ADSC which is in agreement with higher ROS and NO production and down-regulation of antioxidative defence enzyme GPx in these cells. In aged ADSC the mRNA expression level of p53 was down-regulated. On the contrary, in rat and human aged bone marrow derived MSC, we found an increased p53 protein content [44]. Other reported increased p53 gene expression levels in human aged bone marrow MSC [45]. Another factor involved in proliferation but also senescence is c-myc a tumor promoter gene. We found that in aged ADSC c-myc is down-regulated (Figure 1c) as it had been reported for other senescent cell types [46]. The third part of ADSC proliferation is the maintenance of self-renewal, and here we analysed Oct4 expression which is known to be expressed in undifferentiated MSC. We found a small reduction of Oct4 gene expression with age. A loss of stem cell characteristics has been described for MSC from different species and locations. Oct4 expression was also found to be down-regulated in aged mouse satellite stem cells and in hESC cells cultured with serum from old mice [47].

All of these features were accompanied with telomere shortening. This was also for human MSC derived from different age groups [48]. ADSC derived from aged mice showed reduced proliferation potential compared to young ADSC as it was also reported for MSC from bone marrow [44] and did not react to the lower oxygen as was observed for aged rat bone marrow derived MSC [23]. A similar loss of responsiveness to a culture stimulus was observed in aged bone marrow MSC towards a temperature change [49]. It seems that aged MSC are less able to adapt to environmental changes and lose a part of their stem cell potential.

## Angiogenesis during regeneration

MSC can participate in regeneration via paracrine effects. MSC release different factors including growth factors, immune-modulatory factors and chemo-attractants involved in angiogenesis [50]. Angiogenesis is thought to be one of the mechanisms by which MSC can improve heart remodelling [51] or protect from limb ischemia [52]. MSC interact with endothelial cells and support the re-establishment of a blood supply, which is fundamental for tissue repair. Angiogenesis declines with age [53]. Therefore, we compared ADSC derived from young (1-3 months), adult (18 months) and aged (24 months old) mice in their ability to produce angiogenic growth factors.

We found that older ADSC showed impaired angiogenic properties in *in vitro *assays. VEGF and PlGF expression levels in young ADSC exceeded the corresponding levels in aged ADSC. Higher expression of VEGF was also observed in bone marrow derived MSC from young rats compared to 24-26 weeks old rats [23]. Decline of VEGF and TGFb expression as well as significant increase of anti-angiogenic factor TBS2 were found in (6 compared to 24 month) mice. This went along with decreased angiogenesis in these animals [54].

HGF expression increased in aged ADSC. This up-regulation might be explained by a marked autocrine stimulation of ADSC by interleukin 1. Such a mechanism was demonstrated in fibroblasts derived from skin of aged donors [55].

Genes involved in matrix remodelling like uPAR, MMP 2 and MMP 9 were higher in ADSC from 18 month old mice. This point toward an enhanced invasive and migratory capacity of mature progenitors which has not been described before. In the oldest ADSC group, however it declined, which might be linked to impaired repair processes as found in many older organism.

Genes inhibiting angiogenesis like TBS1 and PAI-1 were up-regulated with age. ENDS did not follow this trend. Endostatin, a collagen degradation product, seems to counteract many VEGF induced effects including VEGF induced endothelial migration and neovascularisation [56]. Nothing is known about the age-related secretion of endostatin by mesenchymal stem cells. PAI-1 is a downstream target of p53 and is involved in regulating growth factor signalling [57]. In the liver and adipose tissues of rats PAI-1 was up-regulated in 24 month old rats compared to 3 month old rats [58]. An increase in PAI-1 was also found in senescent human fibroblasts [59].

TBS1, a microcellular protein and an autocrine factor for vascular smooth muscle cells, is constituently expressed in human MSC [60]. TBS-1 is up-regulated in the fibroblasts of 24 month old rats compared to 3 month old rats [61], which correlates with our results in mouse ADSC.

To check the angiogenic effect of the combination of factors secreted by ADSC we used an in vitro tube formation assay, which showed reduced tube formation in the presence of conditioned medium with secreted factors from aged ADSC. Similar results were obtained with bone marrow derived MSC [23] and for young human ADSC [62]. In these studies, however the difference was quite modest.

## Hypoxia and angiogenesis

Tissue damage is often linked to ischemia. That is why it is important to understand the reaction of ADSC towards hypoxia but also because hypoxia can be used to stimulate MSC. Hypoxic conditioning of human ADSC can increase angiogenesis via secreted factors [28,34,63], however nothing is known about this feature in ADSC derived from old donors. The most intensively studied mechanism of cell responses to hypoxia is HIF-1 alpha transcription factor activation. Activation of HIF-1 results in increased gene expression of angiogenesis stimulating factors such as VEGF, angiopoietin, PDGFB, TGFβ_1 _and stromal derived factor 1 [64].

Grafting of hypoxia preconditioned bone marrow derived MSC improves their viability in damaged heart tissue and increases their capacity to stimulate blood vessel growth [65]. In addition hypoxia leads to enhanced MSC migration and formation of capillary-like structures in vitro [66].

No data on the effect of oxygen on the matrix remodelling genes uPAR and uPA in MSC from bone marrow are available. It was shown that gene expression of angiogenesis inhibitors including endostatin and PAI-1 were down-regulated in hypoxic human stromal cells [67] as it was found in our ADSC cells.

In summary, we found that hypoxia stimulated gene expression of pro-angiogenic factors and blocked gene expression of anti-angiogenic factors in ADSC. The effect was smaller in ADSC from older animals. Jiang et al. showed that bone marrow MSC from young rats were superior responders to anoxia both during the acute phase after exposure to anoxia and upon re-oxygenation for 48 hours compared to older rats [23]. Rivard et al. suggested that in aged donors impaired regulation of VEGF in response to hypoxia is due to declined hypoxia inducing factor 1 alpha activity [68].

## Conclusion

Taken together our data shows that aged mice ADSC show impaired proliferation and stem cell characteristics, decreased angiogenic properties and a loss of stimulation by hypoxia suggest that short term hypoxic conditioning of ADSC is still effective in aged animals and might therefore be considered useful in therapies using MSC.

## Competing interests

The authors declare that they have no competing interests.

## Authors' contributions

AE and ES carried out the experimental studies and drafted the graphs. AE, AS and NK participated in the design of the study, performed the statistical analysis and wrote the paper. All authors read and approved the final manuscript.
